# Preliminary analysis of predicting the first recurrence in patients with neovascular age-related macular degeneration using deep learning

**DOI:** 10.1186/s12886-023-03229-0

**Published:** 2023-12-07

**Authors:** Boa Jang, Sang-Yoon Lee, Chaea Kim, Un Chul Park, Young-Gon Kim, Eun Kyoung Lee

**Affiliations:** 1 Department of Transdisciplinary Medicine, Seoul National University Hospital, #101, Daehak-Ro, Jongno-Gu, Seoul, 03080 Republic of Korea; 2 Interdisciplinary Program in Bioengineering, College of Engineering, Seoul National University, Seoul, Republic of Korea; 3 Seoul Shinsegae Eye Clinic, Seoul, Republic of Korea; 4 Department of Medicine, Seoul National University College of Medicine, Seoul, Republic of Korea; 5 Department of Ophthalmology, Seoul National University College of Medicine, Seoul National University Hospital, #101, Daehak-Ro, Jongno-Gu, Seoul, 03080 Republic of Korea

**Keywords:** Anti-VEGF, Deep learning, Neovascular age-related macular degeneration, Optical coherence tomography, Recurrence prediction

## Abstract

**Background:**

To predict, using deep learning, the first recurrence in patients with neovascular age-related macular degeneration (nAMD) after three monthly loading injections of intravitreal anti-vascular endothelial growth factor (anti-VEGF).

**Methods:**

Optical coherence tomography (OCT) images were obtained at baseline and after the loading phase. The first recurrence was defined as the initial appearance of a new retinal hemorrhage or intra/subretinal fluid accumulation after the initial resolution of exudative changes after three loading injections. Standard U-Net architecture was used to identify the three retinal fluid compartments, which include pigment epithelial detachment, subretinal fluid, and intraretinal fluid. To predict the first recurrence of nAMD, classification learning was conducted to determine whether the first recurrence occurred within three months after the loading phase. The recurrence classification architecture was built using ResNet50. The model with retinal regions of interest of the entire region and fluid region on OCT at baseline and after the loading phase is presented.

**Results:**

A total of 1,444 eyes of 1,302 patients were included. The mean duration until the first recurrence after the loading phase was 8.20 ± 15.56 months. The recurrence classification system revealed that the model with the fluid region of OCT after the loading phase provided the highest classification performance, with an area under the receiver operating characteristic curve (AUC) of 0.725 ± 0.012. Heatmap analysis revealed that three pathological fluids, subsided choroidal neovascularization lesions, and hyperreflective foci were important areas for the first recurrence.

**Conclusions:**

The deep learning algorithm allowed for the prediction of the first recurrence for three months after the loading phase with adequate feasibility. An automated prediction system may assist in establishing patient-specific treatment plans and the provision of individualized medical care for patients with nAMD.

**Supplementary Information:**

The online version contains supplementary material available at 10.1186/s12886-023-03229-0.

## Background

Neovascular age-related macular degeneration (nAMD) is a leading cause of blindness in elderly people [1, 2], and the advent of anti-vascular endothelial growth factor (anti-VEGF) therapy has revolutionized the treatment of nAMD [3–5]. Treatment regimens with anti-VEGF agents have relied on retinal fluid in optical coherence tomography (OCT) imaging of the central retinal region to monitor the disease activity and treatment efficacy. The as-needed (pro re nata [PRN]) regimen and the treat-and-extend (TAE) regimen are the two most common strategies used to optimize the management of individual patients. Prediction of disease progression or recurrence using these treatment regimens is especially important in patients with nAMD.

Given the very heterogeneous treatment demand and treatment need of each individual patient, individualized treatment strategies and early detection of recurrence based on changes in pathological fluid seen on OCT are warranted. Kuroda et al. [6] reported that recurrence of retinal exudative change was detected in 65.7% of patients within one year and in 74.8% of patients within two years after the resolution of retinal exudation with initial treatment. Moreover, in cases of severe disease reactivation, if massive subretinal hemorrhage were not treated, irreversible vision loss may occur [7]. Previous studies have reported that retinal thickness and retinal fluid, which include pigment epithelial detachment (PED), subretinal fluid (SRF), and intraretinal fluid (IRF), are common anatomical measures of disease activity in nAMD, and most patients respond well to anti-VEGF agents [8, 9]. Using OCT, clinicians can observe the detailed morphological characterization of macular fluid accumulation. Different morphological changes and the occurrence of retinal fluid are considered important parameters in the prognosis of nAMD [10]. In this study, we will contribute to providing individualized treatment strategies to nAMD patients by predicting the first recurrence after the loading phase.

Recent advances in artificial intelligence, especially deep learning-based convolutional neural networks (CNN), could provide novel promising strategies for the diagnosis of patients with age-related macular degeneration (AMD) [11, 12], and decision-making regarding their treatment [13, 14]. Furthermore, OCT-based response prediction of anti-VEGF treatment, as well as treatment demand in nAMD [15, 16], and visual acuity prediction after initiating treatment [17–19] have shown encouraging results. Nevertheless, to our knowledge, predicting the first recurrence of nAMD after the initiation phase using OCT-based deep learning in nAMD has not yet been investigated, and it is thought that recurrence may be related to quantitative evaluation of retinal thickness and qualitative observation of retinal fluid.

The first three consecutive monthly anti-VEGF injections are generally accepted in clinical practice. However, a consensus has not yet been achieved on when to start the fourth injection after initiating treatment. Depending on reimbursement policies by health insurance systems and physicians' preferences, some physicians may prefer initiating the PRN or TAE regimen after confirming the first recurrence. In contrast, others may prefer the early TAE regimen, which initiates the TAE regimen immediately after three initial doses. Since the timing of the first recurrence is very heterogeneous for individual nAMD patients and the treatment burden caused by overtreatment is high, predicting the first recurrence after initiating treatment is very important. In the current study, we aimed to observe the practical feasibility of a prediction tool using OCT-based deep learning algorithms in patients with nAMD. Specifically, we investigated the feasibility of preliminary analysis of predicting the first recurrence within three months after the initiation of anti-VEGF treatment in a routine clinical setting.

## Methods

### Data sources and participants

We retrospectively reviewed the medical records of 2,266 consecutive patients with treatment-naïve nAMD who visited the Seoul National University Hospital (SNUH) between February 2008 and July 2021. All patients were treated with three consecutive loading intravitreal injections of either ranibizumab (Lucentis; Novartis, Basel Switzerland), aflibercept (Eylea; Bayer Pharma, Germany), or bevacizumab (Avastin; F. Hoffmann-La Roche Ltd, Basel, Switzerland). The study was approved by the Institutional Review Board of Seoul National University Hospital (IRB approval number: 2107–223-1239) and adhered to the tenets of the Declaration of Helsinki. Institutional Review Board of the Seoul National University Hospital waived the need for written informed consent from the participants, because of the study’s retrospective design.

The inclusion criteria were (1) symptomatic nAMD; (2) age ≥ 50 years; (3) three consecutive anti-VEGF injections (i.e., received the first three injections with intervals between each injection shorter than 60 days) followed by PRN dosing; (4) dry macula after the loading phase; (5) availability of both baseline and after the loading OCT images; and (6) follow-up by the time of the first recurrence. The exclusion criteria were (1) other concomitant ocular pathologies that could interfere with visual function; (2) other macular abnormalities (i.e., myopic CNV, angioid streaks or other secondary CNV); (3) persistent exudation despite three consecutive anti-VEGF injections; (4) optical media opacity that substantially disturbed OCT image acquisition; and (5) follow-up loss before the time of the first recurrence.

After initial exclusion, 1,444 eyes from 1,302 patients who met the inclusion criteria were included in this study. Both eyes of the same patient were assessed independently. Before the loading treatment, all patients underwent a comprehensive ophthalmologic examination, including measurement of BCVA, intraocular pressure, slit-lamp biomicroscopy, indirect fundus examination, fundus photography, fluorescein and indocyanine green angiography, and spectral-domain OCT (SD-OCT). Macular 6 × 6 mm OCT scans were obtained using either a Cirrus high-definition OCT (HD OCT, Carl Zeiss Meditec, Dublin, CA, USA) or Spectralis SD-OCT imaging system (Heidelberg Engineering, Heidelberg, Germany). We reviewed the medical records of patients, including demographics, subtypes of nAMD, BCVA, anti-VEGF agents administered, refractive errors, and AL (only in patients with available data). BCVA measurements were made using a Snellen chart and converted to logarithm of the minimum angle of resolution (logMAR) units for statistical analyses. In this study, the first recurrence was defined as the initial appearance of a new retinal hemorrhage or intra/subretinal fluid accumulation after the initial resolution of exudative changes after three loading injections. Although the persistence of PED was not considered a recurrence, the increase in PED size was considered as recurrence. For the first year after the loading phase, monitoring was done every 1 − 2 months, and then every 2 − 3 months until recurrence, depending on the clinician's judgement. Recurrence was evaluated by two independent observers (S.Y.L. and U.C.P.) and a consensus was reached in each case.

### Image preprocessing

Two OCT imaging devices were used to obtain OCT scans. Cirrus OCT scans were acquired at a resolution of 500 × 750 pixels per B-scan, and Spectralis SD-OCT scans were acquired at a resolution of 496 × 768 pixels per B-scan. To obtain a more uniform database, all images were normalized with respect to their horizontal orientation relative to the nose, meaning that images from left eyes were flipped to have the same orientation as the right eyes. Baseline OCT scans were obtained when the patient was first diagnosed with nAMD, whereas OCT scans after the loading phase were taken one month after three consecutive loading injections. Regarding different retinal regions of interest (ROIs), OCT scans of the entire region and fluid region were prepared independently. A scan used for the entire region was resized to 512 × 750 pixels, and a scan cropped by a fluid segmentation model to include retinal fluid was used for the fluid region. The output from the fluid segmentation model was used to determine the center point of the fluid mask, and 400 × 400 patches were cropped using the center point. Then, as an augmentation, these were randomly assigned a value between 0 and 50 to x and y, the coordinate values of the center point, to move the center point and crop the patches. This was proceeded in real time during the learning process.

### Dataset split

All data were randomly divided into training (70%), validation (20%), and test (10%) sets, and both eyes of the same patient were assigned to the same dataset. Considering the potential imbalance of data between the training and test datasets, the target defined in this study was divided into balanced proportions. Five-fold cross-validation (CV) was performed after merging the training and validation sets due to the limited size of the dataset and to prevent overfitting [20]. Five-fold CV was performed by randomly partitioning the data into five subsets of equal size at the patient level. For each CV group, five instances of the recurrence classification model with different random initializations were trained on four subsets and evaluated on one subset. For the final ensemble, the average of the model instances trained in each CV group was used, and the test set was used to evaluate the final performance of each group.

### CNN-based fluid segmentation

A fluid segmentation model was proposed to automatically predict the regions of different fluid compartments, including the PED, SRF, and IRF. A total of 1,105 OCT scans with a fluid mask from the annotated retinal OCT image (AROI) database were used for fluid segmentation. For the AROI database, macular SD-OCT volumes were recorded with the Zeiss Cirrus HD OCT 4000 device [21]. A total of 684 OCT scans collected from SNUH were added as internal data. Fluid segmentation was performed using manual delineation by retinal specialists as the gold standard. Public and internal OCT images was shuffled together and randomly split into 1,220 scans for training, 322 for validation, and 247 for evaluation.

The network architecture was built using U-Net [22]. Standard U-Net architecture was used to identify the three retinal fluid compartments. All the OCT images were resized to 1024 × 512 pixels. The Dice similarity coefficient loss was used to compute the loss between the true mask and the predicted mask in model training [23]. The batch size was set to 8, and an adaptive momentum estimation (Adam) optimizer was applied. The deep learning network was implemented in Python and PyTorch.

### CNN-based recurrence classification

To predict the first recurrence of nAMD, classification learning was conducted to determine whether the first recurrence occurred within three months after the loading phase. The target was defined as a recurrence time interval, and if the first recurrence occurred within three months from one month after three consecutive loading injections, it was defined as a positive target. The recurrence classification architecture was built using ResNet50 [24]. To save training time and directly use diverse underlying features that are difficult to be trained well by a small or specified dataset, transfer learning was used for classification using ImageNet pre-trained weights [25]. Training was performed with a batch size of 16 and 8 for the entire region and fluid region, respectively, and the learning rate was set to 0.0001. Optimization was performed using Adam, and the training loss function for each task was given by the softmax cross-entropy loss between the ground truth label $y$ and the model prediction $\widehat{y}$ given by an input scan $x$. Model training was performed for 100 epochs.

### Evaluation of the predicted model and statistical analysis

The area under the receiver operating characteristic curve (AUC) score was used as a primary metric for predicting the first recurrence of nAMD [26]. In addition, accuracy, sensitivity, specificity, positive predictive value (PPV), negative predictive value (NPV), and F1 score were used. Five-fold CV yielded five different results, so the performance measure was reported using the mean and standard deviation (SD) of the values computed in each fold. The patient-specific predictors of each model were then used as data for a test comparing the two AUCs using the popular area test proposed by DeLong et al. [27], which can determine whether two classifiers have the same AUC score. Kruskal–Wallis and Chi-squared tests were used for comparisons between datasets divided into training and test sets, and a *p*-value of < 0.05 was considered statistically significant. The clinical information was analyzed by dividing into subgroups for each range or subtype, and the comparison of true positive and negative rates between each subgroup was conducted using an analysis of variance (ANOVA).

## Results

### Characteristics of the participants

In total, 1,444 eyes of 1,302 patients were included in the current study (Table 1). Of the 1,302 patients, 716 (54.99%) were male, and 586 (45.01%) were female. The mean age of the patients at baseline was 71.95 ± 8.27 years. The mean logMAR best-corrected visual acuity (BCVA) was 0.71 ± 0.49 at baseline and 0.55 ± 0.48 1 month after the three consecutive loading injections. The axial length (AL) measurement was available for 560 eyes, and the mean AL was 23.54 ± 0.91 mm. Of the 1,444 eyes, 723 (50.07%) were oculus dexters. The nAMD subtypes were as follows: type 1 or 2 choroidal neovascularization (CNV) (1,084 eyes; 75.07%), polypoidal choroidal vasculopathy (PCV, 225 eyes; 15.58%), and retinal angiomatous proliferation (RAP, type 3 CNV, 135 eyes; 9.35%). The anti-VEGF agents administered for three consecutive loading injections were as follows: ranibizumab (659 eyes, 45.64%), aflibercept (638 eyes, 44.18%), and bevacizumab (147 eyes, 10.18%). The mean duration until the first recurrence after the loading phase was 8.20 ± 15.56 months. A total of 888 images from Cirrus and 556 images from the Spectralis OCT system were used. No statistical differences were observed in the demographics and baseline clinical characteristics between the training and test sets.

**Table 1:** Demographics and baseline clinical characteristics of the study participants

Variables	Training	Test	Total	*p*-value
Unique eyes after exclusion applied	1,295	149	1,444	
Gender				
Female	527 (44.97)	59 (45.38)	586 (45.01)	1.00^†^
Age at first nAMD diagnosed (yrs)	71.95 ± 8.27	71.93 ± 8.31	71.95 ± 8.27	0.94^‡^
BCVA at baseline (logMAR)	0.70 ± 0.49	0.76 ± 0.51	0.71 ± 0.49	0.31^‡^
BCVA after loading phase (logMAR)	0.54 ± 0.48	0.58 ± 0.47	0.55 ± 0.48	0.09^‡^
Axial length (mm)^a^	23.54 ± 0.90	23.62 ± 1.03	23.54 ± 0.91	
Oculus				
Oculus dexter	647 (49.96)	76 (51.01)	723 (50.07)	0.88^†^
nAMD subtype				0.26^†^
Type 1 or 2 CNV	969 (74.83)	115 (77.18)	1,084(75.07)	
PCV	208 (16.06)	17 (11.41)	225 (15.58)	
RAP (Type 3 CNV)	118 (9.11)	17 (11.41)	135 (9.35)	
Anti-VEGF used for loading phase				0.55^†^
Ranibizumab	585 (45.17)	74 (49.66)	659 (45.64)	
Aflibercept	576 (44.48)	62 (41.61)	638 (44.18)	
Bevacizumab	134 (10.35)	13 (8.72)	147 (10.18)	
Duration until the first recurrence (months)	7.87 ± 14.38	11.04 ± 23.28	8.20 ± 15.56	0.93^‡^
OCT system				0.84^†^
Cirrus	798 (61.62)	90 (60.40)	888 (61.50)	
Spectralis	497 (38.38)	59 (39.60)	556 (38.50)	

Continuous variables are reported as mean value ± standard deviation. All other data are numbers (percentages)

*nAMD* neovascular age-related macular degeneration, *yrs* years, *BCVA* best-corrected visual acuity, *logMAR* logarithm of the minimum angle of resolution, *anti-VEGF* anti-vascular endothelial growth factor, *CNV* choroidal neovascularization, *PCV* polypoidal choroidal vasculopathy, *RAP* retinal angiomatous proliferation, *OCT* optical coherence tomography

^†^Chi-Square *p*-value

^‡^Kruskal–Wallis *p*-value

^a^Only available in 498 eyes in the training set and 62 eyes in the test set

### CNN-based fluid segmentation

**Fig. 1:**
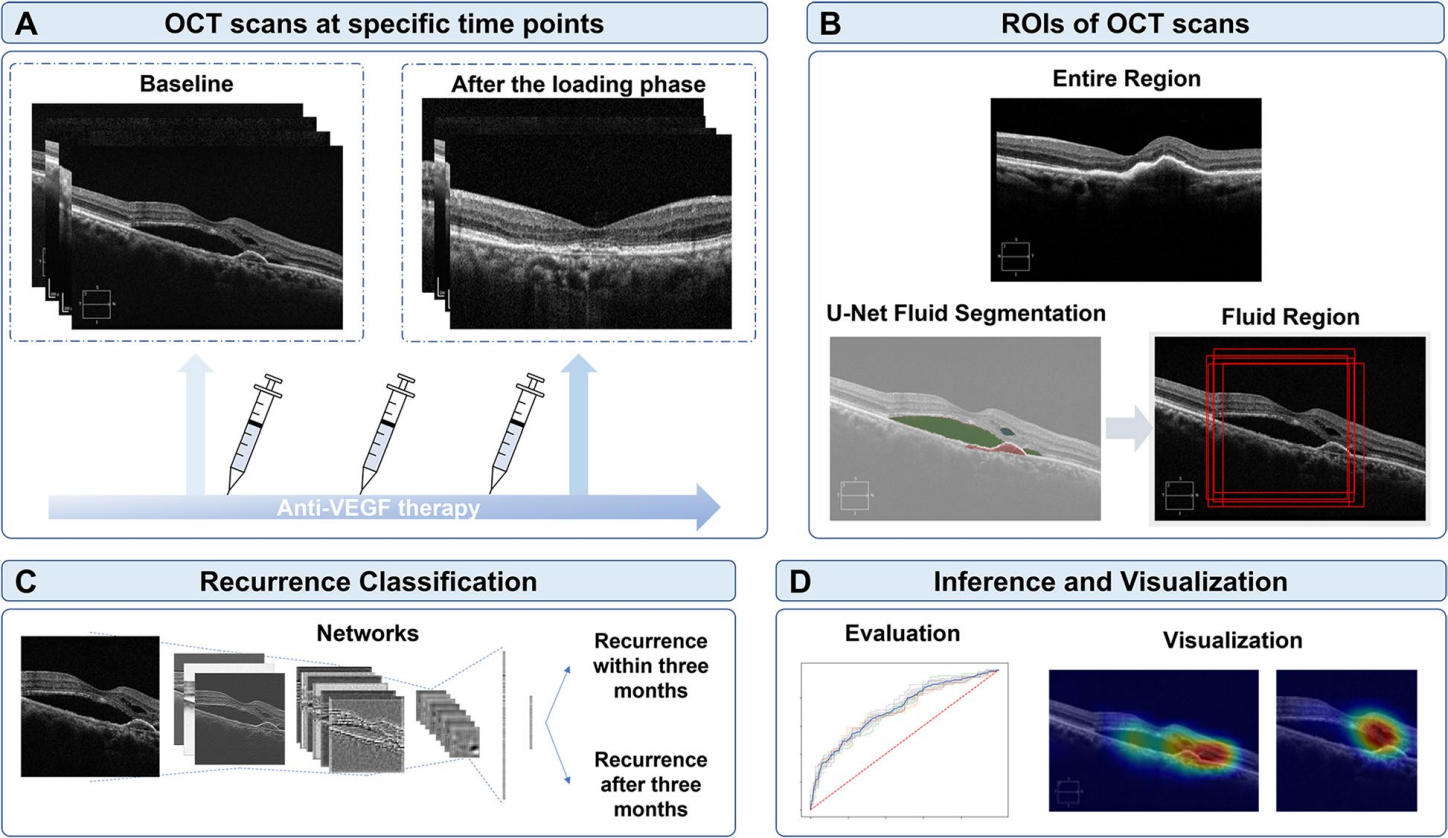
A schematic of deep learning model training, validation, and performance assessment for predicting the first recurrence of neovascular age-related macular degeneration patients using optical coherence tomography (OCT) image sets. **A** OCT scans were obtained at baseline and after the loading phase, which was taken one month after three consecutive anti-vascular endothelial growth factor (anti-VEGF) loading injections. **B** Retinal regions of interest (ROIs) were found using a fluid segmentation network. Recurrence classification network (ResNet50) (**C**) and gradient-weighted class activation mapping visualization (**D**) is presented

**Fig. 2:**
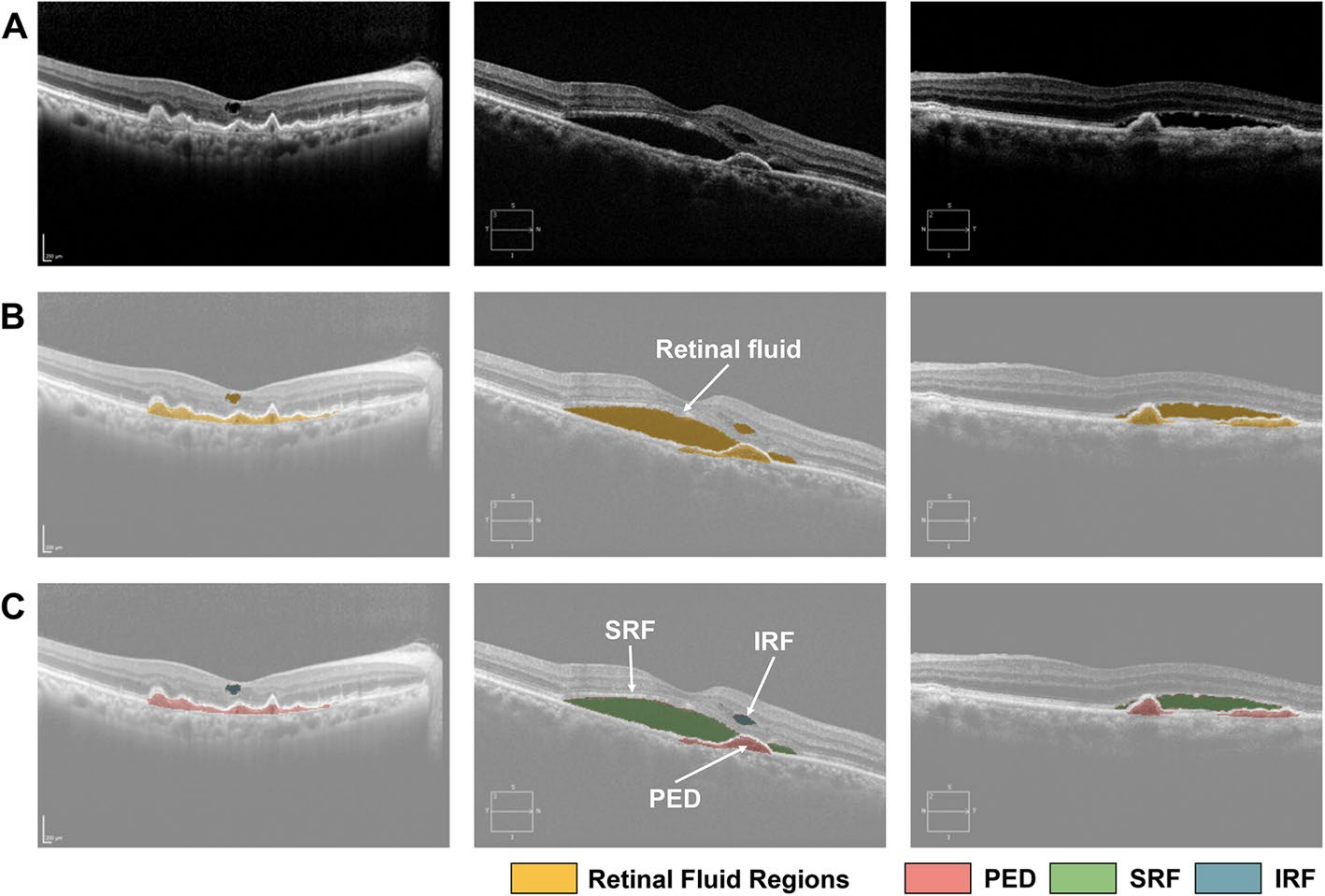
Convolutional neural network-based fluid segmentation results and segmentation color key. **A** Representative original images of optical coherence tomography (OCT) scans. **B** OCT scans with retinal fluid regions. All retinal fluid was coded in yellow. **C** OCT scans with three different retinal fluids. Pigment epithelial detachment (PED) was coded in pink, subretinal fluid (SRF) in green, and intraretinal fluid (IRF) in blue

Two deep learning algorithms were applied to retinal OCT scans: fluid segmentation and recurrence classification (Fig. 1). In total, 247 OCT scans with fluid masks were evaluated for the fluid segmentation model. Using the fluid segmentation algorithm, PED, IRF and SRF segmentations were computed on all OCT volumes for feasibility of quantitative score and retinal region of interest (ROI) for fluid region was extracted for qualitative observations. Examples of segmentations are shown in Fig. 2. Without considering the different types of fluids, all three fluids were considered as one retinal fluid to determine the center point to crop out the fluid region.

### CNN-based recurrence classification

**Fig. 3:**
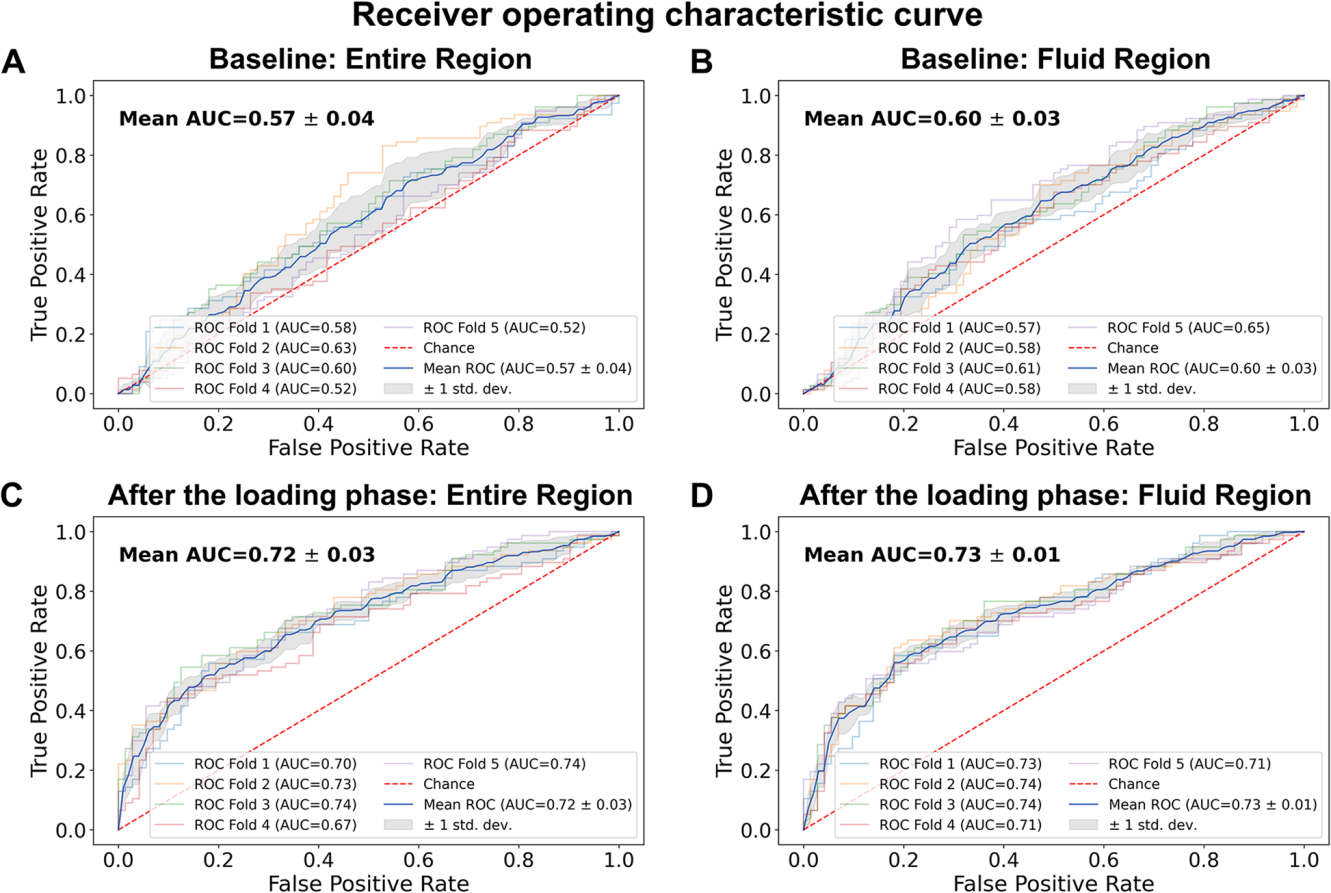
Receiver operating characteristic (ROC) curves for convolutional neural network-based recurrence classification. ROC curves for the entire region (**A**) and the fluid region (**B**) of optical coherence tomography (OCT) scans at baseline. ROC curves for the entire region (**C**) and the fluid region (**D**) of OCT scans after the loading phase

Of a total of 1,444 eyes, after the loading phase, 743 eyes showed the first recurrence within three months and 701 eyes showed the first recurrence after three months. More specifically, the distribution according to the timing of recurrence was 743 (51.45%) within 3 months, 1,034 (71.61%) within 6 months, 1,214 (84.07%) within 1 year, 1,326 (91.83%) within 2 years, and 118 eyes (8.17%) had a first recurrence after 2 years. The distribution of timing of the first recurrence was as follows in each set; Training set (median 2.99 months; interquartile range 1.38 − 6.90; min − max 0.46 − 134.21), Test set (median 2.89 months; interquartile range 1.35 − 7.26; min − max 0.62 − 160.46), Total set (median 2.99 months; interquartile range 1.38 − 7.05; min − max 0.46 − 160.46). A total of 149 eyes from 130 patients in the test set were evaluated at baseline and after the loading phase. Of these eyes, 77 (51.68%) showed the first recurrence within three months, and 72 (48.32%) showed the first recurrence after three months. The results of the recurrence classification task under the four experimental conditions are shown in Table 2 and the results of the receiver operating characteristic (ROC) curve with the area under the receiver operating characteristic curve (AUC) score are shown in Fig. 3. The cutoff point was set to 0.5, which means that any observation with a predicted probability of 0.5 or greater was classified as positive, and any observation with a predicted probability less than 0.5 was classified as negative. The model with the fluid region of OCT scans after the loading phase provided the highest classification performance, with an AUC of 72.5%, followed by the model with the entire region of OCT scans after the loading phase, with an AUC of 71.6%.

**Table 2:** Classification performance of the convolutional neural network models

	**OCT at baseline**		**OCT at after the loading phase**	
	Entire region	Fluid region	Entire region	Fluid region
AUC	0.572 ± 0.043	0.600 ± 0.030	0.716 ± 0.027	0.725 ± 0.012
Accuracy	0.528 ± 0.020	0.572 ± 0.021	0.667 ± 0.016	0.679 ± 0.016
Sensitivity	0.579 ± 0.179	0.566 ± 0.113	0.608 ± 0.083	0.621 ± 0.068
Specificity	0.472 ± 0.172	0.578 ± 0.146	0.731 ± 0.094	0.742 ± 0.075
PPV	0.544 ± 0.02	0.599 ± 0.049	0.717 ± 0.052	0.725 ± 0.036
NPV	0.521 ± 0.035	0.557 ± 0.019	0.638 ± 0.022	0.649 ± 0.022
F1 score	0.545 ± 0.088	0.573 ± 0.039	0.651 ± 0.036	0.665 ± 0.029

*OCT* optical coherence tomography, *AUC* area under the curve, *PPV* positive predictive value, *NPV* negative predictive value

To observe the differences between these four classifiers, the AUC performance between the four classifiers was compared using DeLong’s test. The resulting *p*-values were 0.135 and 0.718, indicating that the estimates do not differ between the entire and fluid regions based on OCT at baseline and after the loading phase, respectively. However, the model comparison results with *p*-values of < 0.001 and 0.009 indicate that the model estimates differ significantly between the baseline and after the loading phase based on the same entire and fluid regions (Table 3). The OCT scan model after the loading phase outperformed the other models in predicting the first recurrence of nAMD.

**Table 3:** Comparison of the AUC performance between four different experimental conditions

Comparison	*p*-value*
Baseline-based entire vs. fluid regions	0.135
After the loading phase-based entire vs. fluid regions	0.718
Entire region-based baseline vs. after the loading phase	**< 0.001**
Fluid region-based baseline vs. after the loading phase	**0.009**
Entire region at baseline vs. fluid region at after the loading phase	**< 0.001**

*vs.* versus, *AUC* area under the curve

^*^DeLong’s test *p*-value. Significant values with *p* < 0.05 are in bold

### Interpretation of the model decisions

The gradient-weighted class activation mapping (Grad-CAM) uses gradients of target to represent a localization map highlighting the main regions in the image for predicting the target [28]. Grad-CAM uses the gradients of any target concept flowing into the final convolutional layer (Conv5_3) of the model trained using OCT scans to extract the feature maps and compute the weights of the feature maps to identify the areas displaying the greatest effect of the first recurrence of nAMD.

**Fig. 4:**
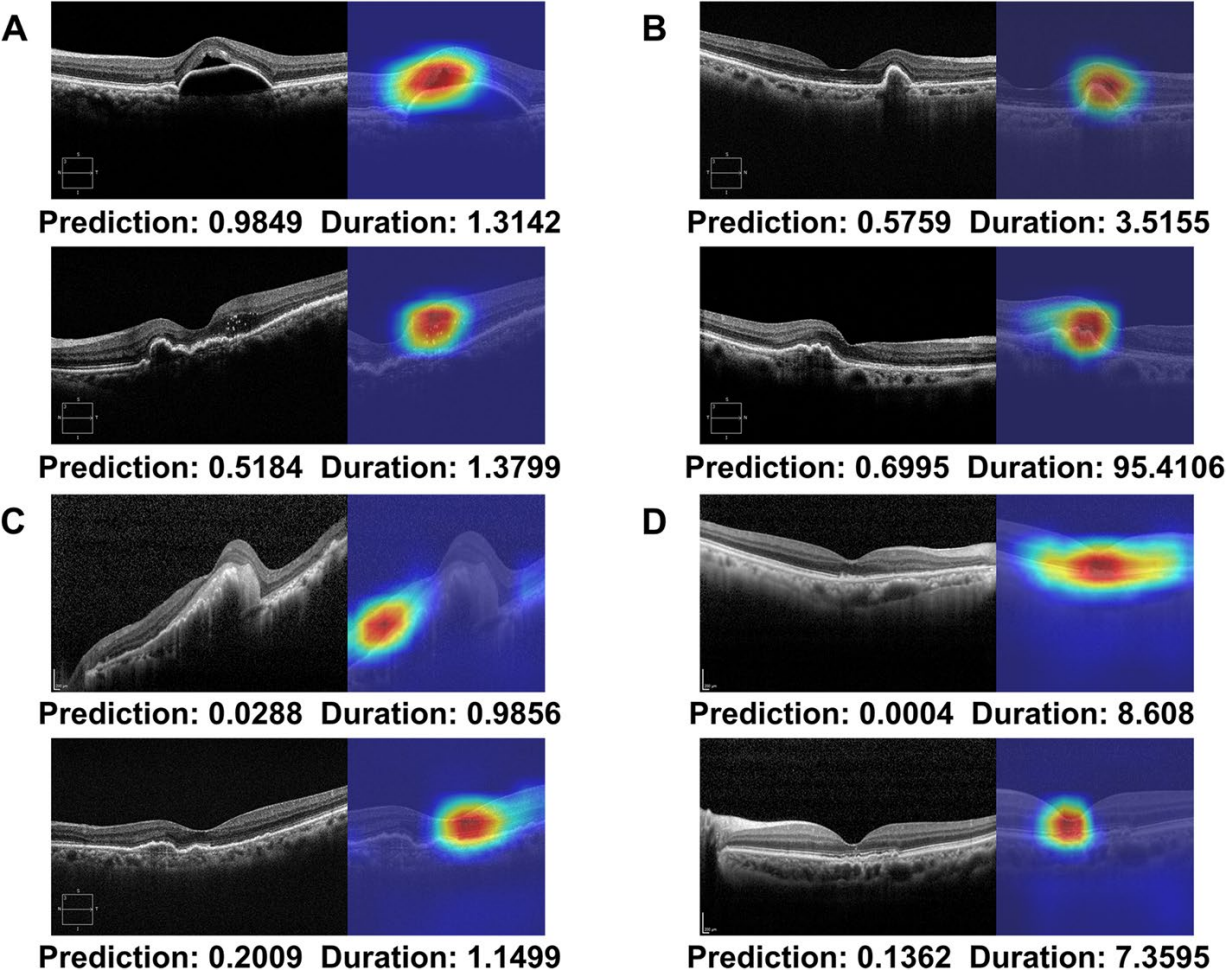
Representative cases of gradient-weighted class activation mapping (Grad-CAM) visualization. Grad-CAM extracts the feature map of the last convolutional layer and shows a heatmap within the image describing the calculated weight of the feature map. **A** True positive; **B** False positive; **C** False negative; **D** True negative cases

**Fig. 5:**
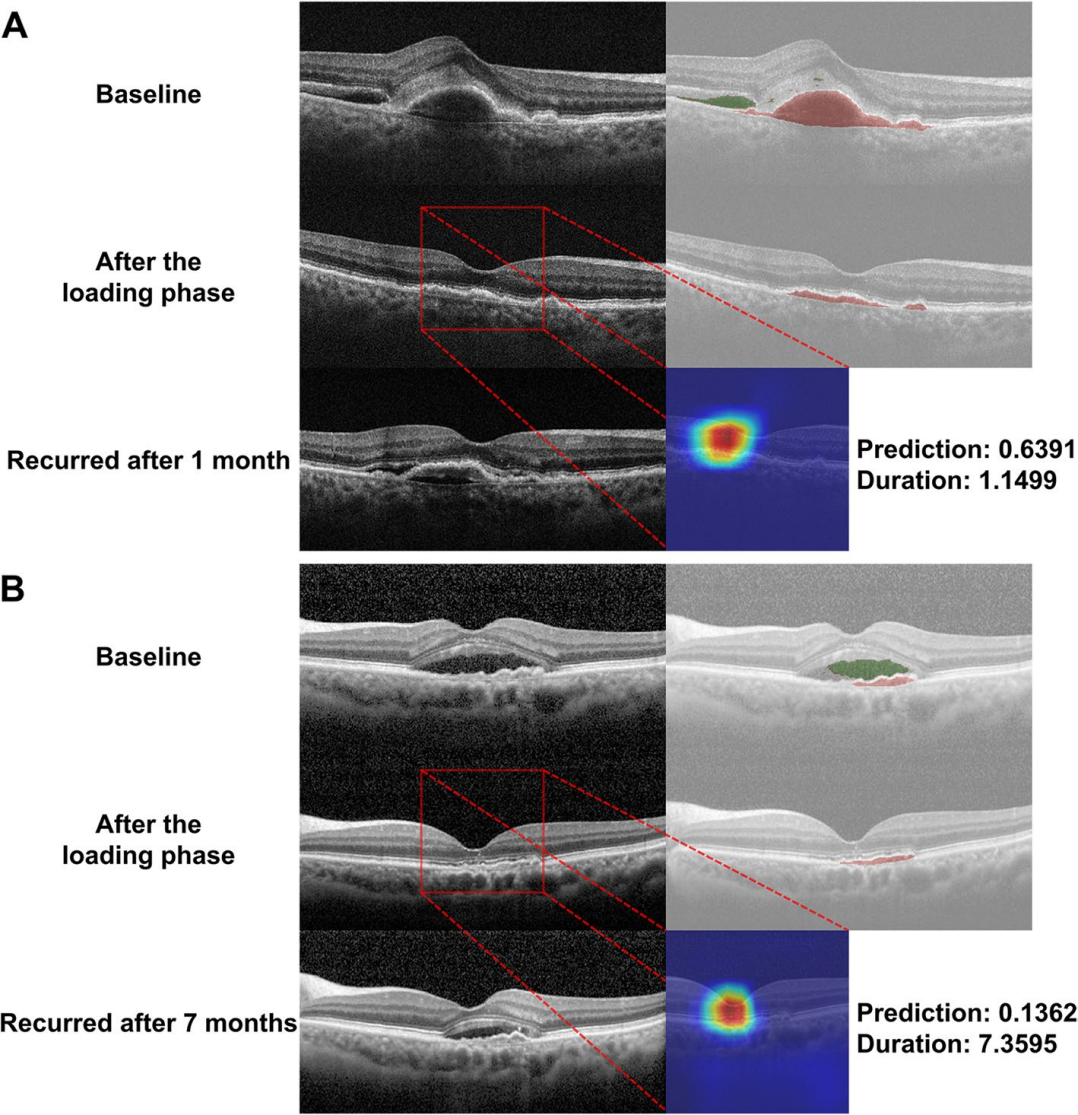
Representative cases of neovascular age-related macular degeneration. Optical coherence tomography (OCT) scans at baseline, after the loading phase, and at the time of the first recurrence are presented. Convolutional neural network-based fluid segmentation results and gradient-weighted class activation mapping (Grad-CAM) are also noted. The red bounding box indicates the area shown by the Grad-CAM. **A** OCT demonstrates the first recurrence at 1.1499 months (within three months) after the loading phase, predicting a value higher than 0.5 with a prediction of 0.6391, which is true positive. **B** OCT demonstrates the first recurrence at 7.3595 months (after three months) after the loading phase, predicting a value lower than 0.5 with a prediction of 0.1362, which is true negative

As shown in Fig. 4, the heatmap represents the most important region in each image of the trained CNN when classified as a recurrence within three months after the loading phase. In true positive cases (Fig. 4A), heatmap analysis with Grad-CAM highlighted areas of IRF and SRF as important areas on the OCT scans. In addition, areas of hyperreflective foci are often highlighted in the OCT images. In false positive cases (Fig. 4B), recurrence occurred after three months, and it was incorrectly predicted that recurrence would occur within three months after the loading phase. Areas of persistent PED are often highlighted on the OCT scans, even though the persistence of PED was not considered to indicate recurrence in the current study. In false negative cases (Fig. 4C), recurrence occurred within three months, and it was incorrectly predicted that recurrence would occur after three months after the loading phase; heatmap analysis concentrated on areas other than the main lesion when there was hemorrhage or large lesion and obtained an incorrect prediction result. There were cases of misprediction when the recurrence occurred very close to the three months’ time point. In true negative cases (Fig. 4D), Grad-CAM mainly highlighted the main lesions of subsided CNV, with one coarse and large-scale attention. Figure 5 shows representative cases of correct predictions of the first recurrence within three months and later.

When classifying the recurrence based on three months, the prediction values were closer to 1 or 0 if the recurrence periods were less than two months or more than four months, respectively, whereas the prediction values were closer to 0.5 if the recurrence periods were close to the three months’ time point (Supplementary Fig. 1).

## Discussion

In the present study, we evaluated the practical feasibility of a prediction tool using OCT-based deep learning algorithms in patients with nAMD. The time to first recurrence of exudation after acquiring a dry macula following three consecutive anti-VEGF loading phase in a routine clinical setting was analyzed to evaluate whether the algorithms could reliably predict the recurrence within three months. Our results demonstrate that model with the fluid region of OCT scans after the loading phase provided the highest classification performance, with an AUC of 72.5%. By proposing a deep learning algorithm to predict the first recurrence using OCT image, we believe that this study has important clinical significance for attempting to individualize decision-making for nAMD patients, which is a heterogeneous disease.

There has been an advance in recent research in AMD utilizing machine learning algorithms, regarding not only diagnosing or classifying diseases but also predicting future events. Schmidt-Erfurth et al. investigated individual disease conversion in early AMD using artificial intelligence [29]. They demonstrated that the model differentiated converting versus non-converting eyes with a performance of 68% and 80% for CNV and geographic atrophy and the most critical features for progression were outer retinal thickness, hyperreflective foci, and drusen area. Ajana et al. evaluated a prediction model for advanced AMD allowing selection of the most predictive risk factors automatically [30]. They revealed that the prediction model achieved an 92% AUC in differentiating the high-risk groups. While it is challenging to make a direct comparison between predicting the progression of early AMD to advanced AMD and predicting the reactivation of nAMD after loading treatment in the current study, the fact that the performance of Schmidt-Erfurth et al.'s model [29] did not exceed 0.8 and the best performance in this study was an AUC of 72.5% shows that it is still challenging to develop a CNN model to predict the future using only OCT images.

Previous studies predicting anti-VEGF treatment demand or frequency in nAMD suggest that machine learning may assist in establishing patient-specific treatment plans in the near future. Gallardo et al. demonstrated mean AUCs of 0.79 and 0.79 for low and high demand in the nAMD-trained models [16], and Pfau et al. revealed mean AUCs from 0.61 to 0.7 for low and high treatment requirement in nAMD patients [31]. Chandra et al. also showed AUCs of 0.79 − 0.82 and 0.79 − 0.81 for predicting few and many injections [32], and Romo-Bucheli et al. revealed AUC of 0.85 in detecting the patients with low and high treatment requirement in nAMD [33]. Meanwhile, machine learning algorithms that predict visual acuity after anti-VEGF therapy may also encourage patients to adhere to intravitreal therapy and contribute to personalized medicine. Rohm et al. evaluated visual acuity at 3 and 12 months in patients with nAMD after initial three anti-VEGF injections and revealed that machine learning allowed visual acuity to be predicted for three months with a comparable result to real visual acuity measurements [17]. Fu et al. investigated the predictive usefulness of quantitative imaging biomarkers from OCT scans in future visual outcomes of nAMD patients starting anti-VEGF therapy [18]. They revealed that visual outcomes under antiangiogenic therapy can be predicted using retinal tissue volumes that have been quantified automatically from OCT images.

In this study, the prediction of recurrence after initial anti-VEGF injections could be used in 3 main ways on an individual patient basis in the form of personalized medicine: (1) It may give caution to high-risk patients who will exhibit early recurrence within three months and encourage them to adhere to regular monitoring. (2) For low-risk patients who are expected to have a late recurrence, it could alleviate the anxieties and follow-up can be more flexible. (3) It could help clinicians in determining the follow-up duration of patients and decision-making around anti-VEGF injections, including recommending or withholding injections.

Although this study did not allow us to determine which biomarkers on OCT were more important features, we did find that the retinal morphological characteristics after the initial three anti-VEGF injections were more important in predicting the recurrence than the retinal appearance with the various pathological fluids at initial presentation. We also found that the clinical significance of pathological fluids on nAMD recurrence still remains crucial, as the model performance was better with fluid ROIs than with entire ROIs of OCT scans. As shown in Figs. 4 and 5, the heatmap results of recurrence classification and retinal fluid segmentation mainly highlighted areas of pathologic fluid, such as PED, SRF, and IRF, as important areas on OCT scans. Grad-CAM highlighted the main CNV lesions or hyperreflective foci to predict early recurrence. The areas of PED were often emphasized in false positive cases, and although persistent PED was not defined as a recurrence in this study, it is well known that PED is also related to CNV activity [34, 35]. Since other pathological fluids, such as SRF and IRF, frequently recur after PED growth, our study may identify that PED is still a meaningful biomarker associated with nAMD recurrence.

Our study has several limitations. First, the number of patients was small, and the recurrence interval was arbitrarily set to three months and classified. Among the patients who experienced recurrence after three months, there was a group of patients who experienced recurrence after 120 months; therefore, further studies are needed to further refine the timing of recurrence. Second, the study population included heterogenous group with different treatment of anti-VEGF agents and treatment interval between each injection. These variations could be confounding factors for prediction of disease recurrence. Nevertheless, we believe that this study is significant in that it evaluates the feasibility of a model to predict recurrence in a real clinical setting where treatment demand and need are highly heterogeneous. Third, it was possible to determine that the retinal fluid had a significant impact; however, it was difficult to determine the relationship between fluid volume and recurrence prediction. Although the performance of the fluid segmentation model was sufficient to detect the fluid region, it may not be accurate to compute the fluid volume on OCT images. Lastly, a series of sequential inputs were not used, but a single OCT image input was utilized in the current study. In the CNN model for time series forecast utilization, OCT images at baseline and after the loading phase could be sequentially utilized. A better model that specifically predicts whether recurrence will occur in an individual patient by combining serial OCT images and clinical information together and the actual time to recurrence is planned in future research.

## Conclusion

In conclusion, we examined the feasibility of predicting the first recurrence within three months after the loading phase of anti-VEGF treatment using OCT-based deep learning algorithms in patients with nAMD in a routine clinical setting. The model with the fluid region of the OCT scans and that after the loading phase provided the highest classification performance. Heatmaps revealed that pathological fluids, such as PED, SRF, and IRF, subsided CNV lesions, and hyperreflective foci were important areas for the first recurrence on OCT scans. This automated prediction system will aid in the provision of individualized medical care for patients with nAMD.

### Supplementary Information

**Additional file 1: Supplementary Figure 1.** Box plot of model prediction between the different groups of recurrence periods. Comparison of the model predicted confidence values for the different recurrence periods, which can be divided into less than two months, within two to four months and more than four months, was performed using an analysis of variance (ANOVA), and the *p*-value is presented above the box plots.

## Data Availability

All data generated or analyzed during this study are included in this published article.

## Abbreviations

nAMD: Neovascular age-related macular degeneration
anti-VEGF: Anti-vascular endothelial growth factor
OCT: Optical coherence tomography
ROIs: Region-of-interests
AUC: Area under the receiver operating characteristic curve
PED: Pigment epithelial detachment
SRF: Subretinal fluid
IRF: Intraretinal fluid
CNV: Choroidal neovascularization
PRN: Pro re nata
TAE: Treat-and-extend
CNN: Convolutional neural network
SNUH: Seoul National University Hospital
BCVA: Best-corrected visual acuity
SD-OCT: Spectral-domain optical coherence tomography
AL: Axial length
logMAR: Logarithm of the minimum angle of resolution
CV: Cross-validation
AROI: Annotated Retinal OCT Images
Adam: Adaptive momentum estimation
PPV: Positive predictive values
NPV: Negative predictive values
ROC: Receiver operating characteristic
SD: Standard deviation
PCV: Polypoidal choroidal vasculopathy
RAP: Retinal angiomatous proliferation
Grad-CAM: Gradient-weighted class activation mapping
