# Supplementary figures and images for: Preliminary analysis of predicting the first recurrence in patients with neovascular age-related macular degeneration using deep learning
Source: BMC Ophthalmol. 2023 Dec 7;23:499. doi: 10.1186/s12886-023-03229-0 (PMC10702052; doi:10.1186/s12886-023-03229-0)

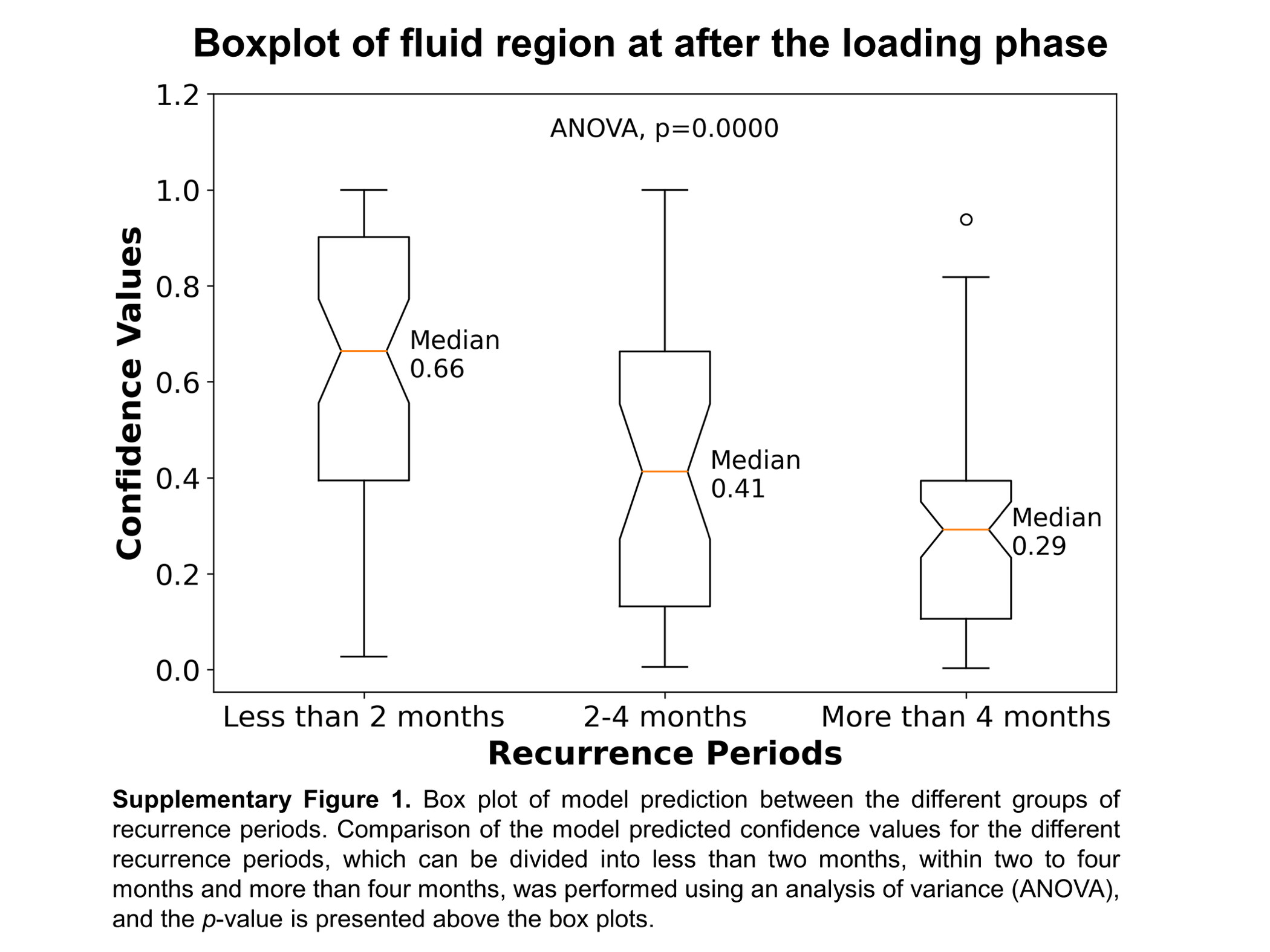

Supplement: Supplementary file 1 — Additional file 1: Supplementary Figure 1. Box plot of model prediction between the different groups of recurrence periods. Comparison of the model predicted confidence values for the different recurrence periods, which can be divided into less than two months, within two to four months and more than four months, was performed using an analysis of variance (ANOVA), and the p-value is presented above the box plots. [file 12886_2023_3229_MOESM1_ESM.tif]
